# 沉默血管生成素-2表达对人肺腺癌细胞生物学特性的影响

**DOI:** 10.3779/j.issn.1009-3419.2011.07.01

**Published:** 2011-07-20

**Authors:** 白翎 李, 冠鑫 张, 霄雷 侯, 扬 袁, 晓红 刘, 德军 龚, 盛东 黄

**Affiliations:** 1 200433 上海，第二军医大学附属长海医院胸心外科 Department of Cardiothoracic Surgery, Changhai Hospital, Second Military Medical University, Shanghai 200433, China; 2 200437 上海，上海中医药大学附属岳阳中西医结合医院重症监护室 Department of ICU, Yueyang Hospital of Integrated Traditional Chinese and Western Medicine, Shanghai 200437, China

**Keywords:** 血管生成素-2, A549细胞株, 腺相关病毒, RNA干扰, Angiopoietin-2, A549 cell strain, Adeno-associated virus, RNA interference

## Abstract

**背景与目的:**

促血管生成素-2（Ang-2）是一类调节血管生成的重要细胞因子，与肿瘤的发生发展有密切的联系。本研究通过腺相关病毒（adeno-associated virus, AAV）介导的RNA干扰（RNA interference, RNAi）抑制肺腺癌细胞A549 Ang-2的表达，观察其对癌细胞生物学特性的影响。

**方法:**

构建H1启动子驱动的表达针对Ang-2的小干扰RNA（siRNA）重组腺相关病毒（AAV-Ang-2^shRNA^），转染A549细胞，同时以正常A549细胞及其转染空病毒（AAV-Null）的A549细胞作为对照，观察RNAi沉默Ang-2表达在体外及体内对A549细胞生长、致瘤、增殖、凋亡及肿瘤微血管密度的影响。

**结果:**

体外实验结果表明重组腺相关病毒转染A549细胞48 h后Ang-2蛋白表达比对照组明显降低（*P* < 0.001）；细胞生长明显减慢（*P* < 0.001）。RNA干扰后A549细胞细胞周期分析结果提示A549细胞对照组、AAV-Null对照组和AAV-Ang-2^shRNA^实验组的增殖指数（proliferation index, PI）分别为0.51±0.43、0.48±0.29和0.26±0.31。AAV-Ang-2^shRNA^实验组与对照组比较，PI明显减少（*P*=0.001)。体内实验结果表明，AAV-Ang-2^shRNA^实验组在胸腺缺陷小鼠成瘤体积、瘤质量均明显低于两对照组（*P* < 0.01）。各组微血管密度分析结果分别为46.4±13.1、44.2±13.6和34.9 ±12.8；AAV-Ang-2^shRNA^实验组肿瘤组织内新生血管数目明显低于对照组（*P* < 0.001）。各组凋亡指数（apoptosis index, AI）分别为（5.98±3.11）%、（7.51±4.42）%及（17.06±7.43）%。AAV-Ang-2^shRNA^组比PBS组、AAV-Null组凋亡百分率明显增高（*P*=0.005, *P*=0.007）。各组细胞增殖核抗原（proliferating cell nuclear antigen, PCNA）阳性表达率分别为（92.75±9.7）%、（85.8±11.8）%、（69.8±16.5）%；AAV-Ang-2^shRNA^组PCNA指数低于两对照组（*P*=0.014, *P*=0.021）。

**结论:**

腺相关病毒介导的siRNA表达能明显抑制A549细胞中Ang-2的表达，减慢肿瘤细胞的增殖，促进肿瘤细胞凋亡，抑制肿瘤生长。

## Abstract (en)

**Background and objective:**

It is well-known that angiopoietin-2 (Ang-2) plays an important role in the formation of the blood vascular system. Angiopoietin is involved in many diseases about angiogenesis such as tumor, so may have great prospects for the treatment of these diseases. The aim of this study is to evaluate the influence of inhibiting Ang-2 via adeno-associated virus induced RNA interference (RNAi) on the biological characteristics of bronchogenic adenocarcinoma.

**Methods:**

AAV-Ang-2^shRNA^ driven by H1 promoter was constructed to transfect A549 cell line. Normal and AAV-Null cell line were utilized in the control groups. The influence of RNAi on Ang-2 expression as well as the growth rate, tumorigenic efciency, proliferation rate, apoptosis, and microvessel density of A549 cell line were analyzed.

**Results:**

*In vitro* experiment indicated that the Ang-2 expression level (*P* < 0.001) and growth rate (*P* < 0.001) of A549 cell line 48 h transfected with AAV-Ang-2^shRNA^ were signifcantly lower than those in the normal and AAV-Null cell lines. Cell cycle analysis showed the proliferation index (PI) of normal, AAV-Null, and AAV-Ang-2^shRNA^ transfected A549 cell line were 0.51±0.43, 0.48±0.29, and 0.26±0.31, respectively, which indicated the PI of AAV-Ang-2^shRNA^ transfected cell line was signifcantly lower, compared with the normal and AAV-Null cell lines. *In vivo* experiment exhibited that AAV-Ang-2^shRNA^ transfected cell line possessed a lower mass and volumn of tumor relative to two control groups. In addition, the apoptosis index (AI) of AAV-Ang-2^shRNA^ transfected, normal, and AAV-Null cell lines were (5.98±3.11)%, (7.51±4.42)% and (17.06±7.43)% respectively, which manifested that AAV-Ang-2^shRNA^ transfected cell line possessed a higher AI (*P*=0.005, *P*=0.007). A lower percentage of PCNA-positive cell was observed in AAV-Ang-2^shRNA^ transfected cell line (92.75±9.7)% as well, compared with the normal (85.8±11.8)% and AAV-Null (69.8±16.5)% cell lines.

**Conclusion:**

AAV-mediated expression of shRNA signifcantly reduces concentration of Ang-2 in A549 cell line, lowers proliferation and growth rate and induce.apoptosis of A549 cell line.

肺癌是我国发病率最高的恶性肿瘤之一，侵袭和转移是其最本质的生物学特征，也是导致肺癌死亡的根本原因。血管新生是体内实体瘤生长和转移的重要环节，恶性肿瘤的侵袭、转移均与肿瘤血管生成密切相关^[1, 2]^。血管生成素-2（Angiopoietin, Ang-2）蛋白表达是肿瘤血管新生起始的强化因素，与肿瘤血管生成的数目、肿瘤的大小和肿瘤的侵袭转移密切相关^[3, 4]^。但以Ang-2为靶点的基因靶向治疗，在肺腺癌研究中未见相关报道。本研究采用针对Ang-2的siRNA重组腺相关病毒AAV-Ang-2^shRNA^，转染肺腺癌细胞A549；通过沉默Ang-2表达观察癌细胞生物学特性的变化，探讨Ang-2在肺腺癌发生过程中的作用，为肺腺癌抗血管生成疗法奠定基础。

## 1 材料与方法

### 1.1 实验材料

重组腺相关病毒AAV-Ang-2^shRNA^由中国人民解放军胸心外科研究所制备完成，并用于后续实验。人肺腺癌细胞株A549购自中科院上海细胞生物学研究所。SPF级BALB/C4-6周龄雌性裸鼠由中国人民解放军第二军医大学实验动物中心提供。小鼠抗人Ang-2单抗购自美国Santa Cruz公司。

### 1.2 重组腺相关病毒AAV-Ang-2^shRNA^转染肺腺癌细胞A549体外实验

AAV转染肺腺癌细胞A549：24孔培养板培养A549细胞至密度约80%时，根据随机对照表，取3孔细胞计数，按感染复数（MOI值）100加入重组腺相关病毒AAV-Ang-2^shRNA^（实验组），取3孔细胞计数后按MOI值100加入空腺相关病毒AAV-Null（对照组），另取3孔细胞计数后不加腺相关病毒（空白对照组）。

#### 1.2.1 Western blot法检测转基因A549细胞中Ang-2蛋白表达

分别收集培养48 h后的正常A549细胞、AAV-Null转染细胞及AAV-Ang-2^shRNA^转染细胞，5%SDS裂解细胞，细胞裂解上清液经十二烷基磺酸钠聚丙烯酰胺凝胶电泳，转印至硝酸纤维素膜上，经过小鼠抗人Ang-2单抗（美国Santa Cruz公司）和羊抗小鼠IgG-HRP多抗两步免疫反应，免疫化学发光显色，以β-actin为内参照，检测3组细胞Ang-2蛋白表达量。

#### 1.2.2 ELISA法检测RNA干扰对A549细胞分泌Ang-2蛋白的影响

分别于转染后1 d、2 d、3 d、4 d、5 d收集各组培养上清，用10%的小牛血清1640培养液作对照组（mock infection）。在波长450 nm纠正波长570 nm条件下读取光密度值，样品浓度从相应标准曲线查得。

#### 1.2.3 MTT法检测细胞增殖

细胞接种于96孔板后，分别于接种后的1 d、2 d、3 d、4 d、5 d加入20 μg MTT（5 g/L）/孔，4 h弃上清，加二甲基亚砜150 μL，振荡10 min至紫色结晶完全溶解，酶标仪490 nm波长处测各孔光密度值（OD值）。试验重复3次，绘制细胞生长曲线。

#### 1.2.4 流式细胞检测RNA干扰后A549细胞周期

分别收集A549细胞组、AAV-Null组及AAV-Ang-2^shRNA^组的对数生长中期细胞，按常规方法进行细胞周期检测，计算细胞的增殖指数（proliferation index, PI）：PI=(S+G_2_)/(S+G_2_+G_1_)。

### 1.3 重组腺相关病毒AAV-Ang-2^shRNA^转染肺腺癌细胞A549体内实验

裸鼠成瘤实验：实验裸鼠随机分为3组，每组6只：AAV-Ang-2^shRNA^实验组、AAV-Null空病毒对照组、PBS缓冲液空白对照组。在裸鼠背部皮下注射对数生长期A549细胞0.2 mL（2×10^7^个）。当裸鼠皮下形成种植瘤后（约在A549注射1周左右出现肉眼可见的瘤体），用1 mL注射器抽取7.0×10^7^ IU/mL的AAV-Ang-2^shRNA^靶向重组腺相关病毒100 μL，在肿瘤长径注射点处多方向瘤内注射，对照组注射等量的PBS液、空病毒。隔日1次，共5次。接种30 d后测量形成肿瘤的长度（L）和宽度（W），依据公式计算肿瘤体积（V）=0.5×L×W^2^。处死小鼠，取出瘤体称重。

#### 1.3.1 Ang-2、VEGF表达检测

肿瘤标本均经10%中性福尔马林固定，石蜡包埋。常规制备5 μm-6 μm厚连续切片3张，用于免疫组化染色。兔抗人Ang-2、VEGF多克隆抗体、即用型SABC免疫组化染色试剂盒、DAB显色试剂盒购自武汉博士德公司。免疫组化染色采用SABC法，高温抗原修复，染色程序参照说明书进行。用已知阳性切片作为阳性对照，PBS置换一抗作为阴性对照。每张切片随机取6个视野（×400）。阳性细胞数 < 5%为阴性，5%-24%为弱阳性（+），25%-50%为中等强度阳性（++）， > 50%为强阳性（+++）。

#### 1.3.2 微血管密度（microvessel density, MVD）计数

3.7%的中性甲醛溶液固定肿瘤组织，石蜡切片，0.3%过氧化氢溶液室温下孵育10 min，DAB显色，苏木精衬染，在光镜下行微血管密度计数。在200倍镜下（200×0.785 mm^2^/视野）计数5个热点区的血管数，取其平均值作为该只裸鼠肿瘤的MVD。取同一组3只裸鼠肿瘤的平均MVD，作为该组的平均MVD。

#### 1.3.3 原位凋亡染色（TUNEL法）

常规石蜡切片脱蜡至水后用PBS冲洗3 min×3次。20 ng/mL新配制的蛋白酶K在37 ℃下消化20 min；0.1 mmol/L甘氨酸-PBS洗5 min，PBS洗3 min×3次。加入30 μL的TUNEL反应液，37 ℃作用60 min，PBS洗3 min×3次。甘油明胶封片，显微镜下观察，计数细胞凋亡指数。

#### 1.3.4 细胞增殖核抗原（proliferating cell nuclear antigen, PCNA）免疫组化染色

采用免疫组化ABC法检测，操作步骤如下：新鲜肿瘤组织用10%甲醛固定，丙酮逐级脱水，石蜡包埋切片，脱蜡后按免疫组化ABC法进行免疫组织化学染色、制片。以PBS液代替一抗作阴性对照。

### 1.4 统计学处理

采用SPSS 13.0软件分析系统处理结果，计量资料采用Mean±SD表示，计数资料用率表示，应用*t*检验、方差分析或*χ*^2^检验分析数据，以*P* < 0.05为差异有统计学意义。

## 2 结果

### 2.1 靶向RNA干扰A549细胞后Ang-2蛋白表达

AAV-Ang-2^shRNA^组、AAV-Null组和A549组的Ang-2蛋白条带灰度扫描值分别为370.47±64.99、907.86±95.59和1, 019.21±72.24。可见A549细胞转染AAV-Ang-2^shRNA^后48 h，Ang-2蛋白表达较AAV-Null转染组、A549对照组明显降低（*P* < 0.001）（图 1）。

**1:**
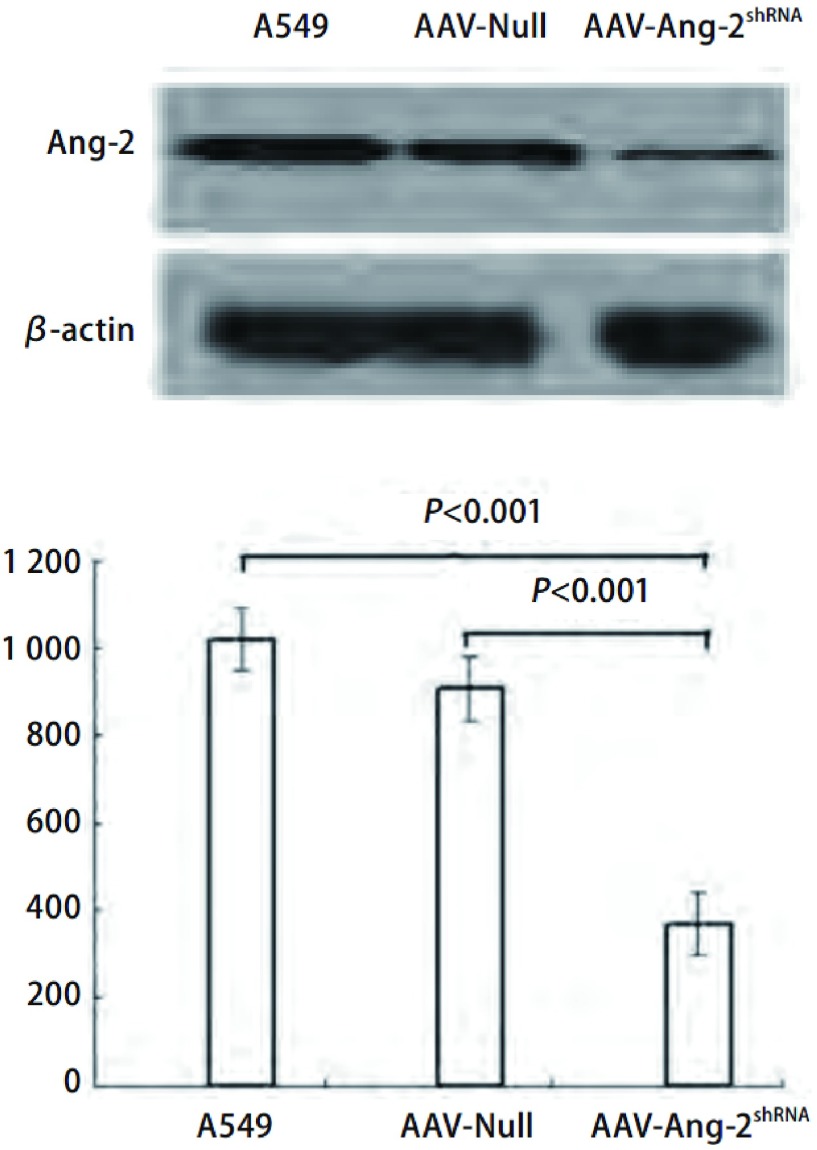
Western blot检测Ang-2蛋白的表达 Western blot detected the expression of Ang-2 protein

### 2.2 靶向RNA干扰A549细胞后ELISA检测Ang-2分泌情况

A549组与AAV-Nul l组Ang-2表达无统计学差异（*P* > 0.05），AAV-Ang-2^shRNA^转染A549细胞其培养液中Ang-2表达分别与A549组及AAV-Null组比较明显降低（*P* < 0.001）（表 1）。

**1:** ELISA法检测Ang-2的表达（pg/mL） The expression of Ang-2 detected by ELISA (pg/mL)

Group	1 d	2 d	3 d	4 d	5 d
A549	44.7±6.43	88.9±5.86	154.4±8.96	206. 5±3.65	211.9±5. 38
AAV-Null	36.9±11. 36	85.0±8. 21	147.6±10.48	212.4±7. 54	227.7±7. 59
AAV-Ang-2^shRNA^	38.1±8.14	38.2±3. 55^*△^	54.5±6.33^*△^	67.7±5. 24^*△^	73.6±8.42^*△^
*SNK* test: compared with the A549 group, ^*^P < 0.01; compared with AAV-Null group, ^△^*P* < 0.01.					

### 2.3 靶向RNA干扰后MTT检测细胞生长情况

AAV-Ang-2^shRNA^实验组细胞生长曲线较平缓，细胞生长速度较A549组、AAV-Null组明显降低（表 2）。统计学分析提示各组随时间变化趋势有统计学意义；SNK检验提示：第1、2日各组间无统计学差异（*P* > 0.05）。3日后各组间比较有统计学意义。其中，两对照组间OD值比较无统计学差异（*P* > 0.05）；AAV-Ang-2^shRNA^组分别与A549组、AAV-Null组比较，OD值明显减少，差异有统计学意义（*P* < 0.01）。以上结果表明，靶向Ang-2 RNA干扰使肿瘤细胞增殖明显减慢。

**2:** MTT检测RNA干扰后Ang-2表达 Expression of Ang-2 after treatment with RNAi dectected by MTT

Group	1 d	2 d	3 d	4 d	5 d
A549	0.57±0.03	0.95±0.05	1.44±0.01	1.71±0.05	1.76±0.01
AAV-Null	0.55±0.02	0.96±0.03	1.45±0.05	1.72±0.05	1.77±0.01
AAV-Ang-2^shRNA^	0.56±0.02	0.95±0.01	1.09±0.01^*△^	1.24±0.13^*△^	1.28±0.09^*△^
*SNK* test: compared with the A549 group, ^*^P < 0.01; compared with AAV-Null group, ^△^*P* < 0.01.					

### 2.4 靶向RNA干扰后流式细胞检测细胞周期分析

A549细胞组、AAV-Null组和AAV-Ang-2^shRNA^组PI分别为0.51±0.43、0.48±0.29和0.26±0.31。可见AAV-Ang-2^shRNA^组与A549组、AAV-Null组比较，PI明显降低（*P*=0.001, *P*=0.001）（图 2）。

**2:**
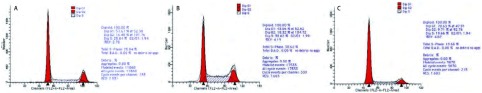
流式细胞检测RNA干扰后细胞周期变化。A：A549组；B：AAV-Null组；C：AAV-Ang-2^shRNA^组。 Flow cytometry cell cycle after RNA interference. A: A549 group; B: AAV-Null group; C: AAV-Ang-2^shRNA^ group.

### 2.5 裸鼠体内成瘤实验

接种30 d后处死裸鼠，取瘤称重。PBS组、AAV-Null组和AAV-Ang-2^shRNA^组瘤体积分别为（709.6±140.5）mm^3^、（691.83±76.7）mm^3^、（484.3±114.6）mm^3^，质量分别为（2.21±0.17）g、（1.98±0.29）g、（1.38±0.35）g，PBS组和AAV-Null空病毒组质量及体积比较差异无统计学意义（*P* > 0.05）。而AAV-Ang-2^shRNA^组与PBS组和AAV-Null组比较，体积明显缩小（*P*=0.001, *P*=0.003），质量减轻（*P*=0.003, *P*=0.004）。结果表明，AAV-Ang-2^shRNA^组肿瘤生长速度明显低于AAV-Null组和PBS组。

### 2.6 Ang-2、VEGF的表达检测

Ang-2蛋白阳性染色均定位于胞浆内，呈现粗细颗粒状棕黄色着色。VEGF定位于肿瘤细胞的细胞浆或细胞膜；阳性物质均呈棕黄色（图 3）。结果显示，AAV-Ang-2^shRNA^实验组、A549组、AAVNull组的Ang-2阳性表达率分别为（11±3）%、（77±9）%及（83±12）%；实验组与对照组比较差异有明显统计学意义（*P* < 0.001, *P* < 0.001），AAV-Ang-2^shRNA^组、A549组、AAV-Null组VEGF阳性表达率分别为（69±17）%、（73±11）%及（81±8）%；各组比较差异无统计学意义（*P* > 0.05）。表明Ang-2靶向RNA干扰后肿瘤组织内Ang-2表达量明显降低，但对VEGF的表达没有明显的影响。

**3:**
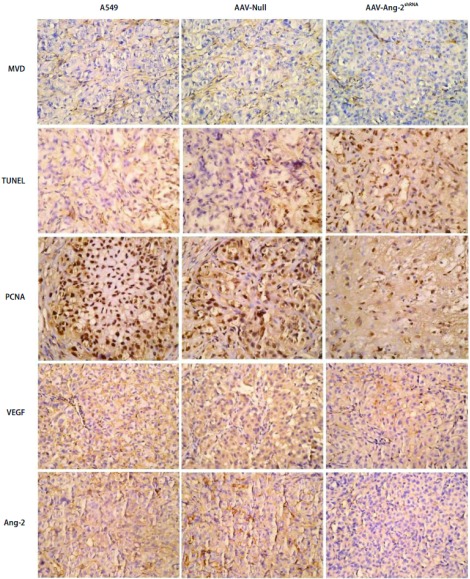
Ang-2靶向RNA干扰A549细胞后免疫组化分析（×400）。MVD计数：FVⅢ因子染色定位于血管内皮细胞，呈棕黄色；TNNEL染色：TdT酶介导的原位末端标记法，凋亡细胞核呈棕黄色；PCNA计数：阳性表达定位于细胞核，显示为棕黄色。Ang-2蛋白阳性染色均定位于胞浆内，呈现粗细颗粒状棕黄色着色。VEGF定位于肿瘤细胞的细胞浆或细胞膜；阳性物质均呈棕黄色。 Ang-2 RNA interference targeting A549 cells immunohistochemical analysis (×400). MVD count: FVⅢ factor was localized in vascular endothelial cells, brownish yellow; TNNEL staining: TdT-mediated in situ end labeling of apoptotic nuclei showed brown; PCNA count: expression located in the nucleus, appears as brown. Ang-2 protein positive staining were localized in the cytoplasm, showing the thickness of granular brown staining. VEGF localized in the cytoplasm of tumor cells or the cell membrane; positive material showed a brown-yellow.

### 2.7 MVD计数结果

PBS组、AAV-Null组和AAV-Ang-2^shRNA^组MVD计数分别为46.4±13.1、44.2±13.6和34.9±12.8（图 3）。结果显示，AAV-Ang-2^shRNA^组分别与A549组和AAVNull组比较差异有明显统计学意义（*P* < 0.001, *P* < 0.001），而PBS组和AAV-Null组比较差异无统计学意义（*P* > 0.05)，表明AAV-Ang-2^shRNA^组肿瘤组织内新生血管数目明显低于AAV-Null组和PBS组。

### 2.8 原位凋亡染色计数细胞凋亡指数（apoptosis index, AI）（TUNEL法）

每组选取12张切片，每张切片取3个阳性视野观察并计数500个细胞，PBS组、AAV-Null组、AAV-Ang-2^shRNA^组凋亡指数分别为（5.98±3.11）%、（7.51±4.42）%及（17.06±7.43）%。结果显示，AAV-Ang-2^shRNA^组肿瘤细胞凋亡明显多，呈簇状分布，而对照组仅有散在差异分布的凋亡细胞（图 3），PBS组、AAV-Null组凋亡指数比较无统计学意义（*P* > 0.05）；AAV-Ang-2^shRNA^组比两对照组凋亡指数明显增加，差异有统计学意义（*P*=0.005, *P*=0.007）。

### 2.9 PCNA检测

PCNA阳性表达定位于细胞核，显示为棕黄色（图 3）。每组动物观察12张切片，每张切片取3个视野，计算肿瘤细胞PCNA指数（PCNA label index, PCNA LI），取其均值。PCNA LI=阳性细胞数/肿瘤细胞数×100%。各组PCNA阳性表达率分别为（92.75±9.7）%、（85.8±11.8）%、（69.8±16.5）%。统计结果显示，PBS组、AAV-Null组间PCNA指数比较差异无统计学意义（*P* > 0.05），AAV-Ang-2^shRNA^组比PBS组、AAV-Null组PCNA指数明显降低，差异有统计学意义（*P*=0.014, *P*=0.021）。

## 3 讨论

肿瘤的生长主要依赖血管的供应，同时肿瘤血管也是肿瘤细胞向其它组织和器官转移的重要途径^[5]^。作为血管生成因子的家族，血管生成素在肿瘤血管生成和肿瘤行为方面起着独特的作用。作为血管生成素家族的成员，Ang-2尤其成为了研究的焦点。Ang-2作为Ang家族的成员之一，是一种分泌型糖蛋白，位于人类染色体的8p23上，相对分子质量接近75 kDa。Ang-2是一种特异性血管生成刺激因子，也是恶性肿瘤早期的标志分子^[6]^。近年来的研究^[7]^表明Ang-2与肿瘤本身的生长和侵袭转移及肿瘤性血管的生成密切相关，Ang-2主要参与新生血管的成熟及维持血管稳定性，是肿瘤血管新生的关键因素。在肿瘤发生的早期，Ang-2参与破坏瘤体周围宿主的正常血管而促进肿瘤新生血管的生成，在瘤体周边形成所谓的血管共择区^[8]^。当肿瘤形成以后，Ang-2通过打断内皮细胞和内皮周围细胞的相互作用来促进血管生芽和血管去稳定，从而增强了VEGF的刺激作用，因此Ang-2与VEGF有协同作用，共同促进肿瘤血管生成，并阻碍血管的成熟性和稳定性^[9, 10]^。

近期研究^[11, 12]^发现，在肺腺癌等肿瘤组织中Ang-2存在高表达，提示以Ang-2为靶点进行抗肿瘤血管新生治疗的可能性。Morrissey等^[13]^发现，Ang-2在前列腺癌的血管新生中作用明显，它特异表达于肿瘤组织的血管重建区并参与肿瘤血管的新生，从而影响肿瘤的生长和转移，并且下调Ang-2的表达可以抑制前列腺癌细胞的生长。Pietrowski等^[14]^研究报道，Ang-2通过拮抗Ang-1的作用破坏肿瘤血管，消除血管基底膜和血管旁细胞对血管形成的限制，并活化内皮细胞，促进肿瘤的增殖、侵袭、迁徙。Kikuchi等^[15]^对卵巢癌细胞的相关研究发现，抑制Ang-2的表达可以明显降低卵巢癌细胞的增殖，促进细胞凋亡。

本实验通过RNA干扰技术，采用腺相关病毒为载体，将外源性siRNA Ang-2基因转入人肺腺癌细胞系A549中。运用Western blot及ELISA法检测RNA干扰后Ang-2蛋白表达及分泌情况。结果显示，Ang-2表达在转染后明显降低，提示通过腺相关病毒为载体能将siRNA Ang-2高效转入肺腺癌细胞中，获得满意的RNA干扰效果。我们进一步采用MTT法观察AAV-Ang-2^shRNA^转染的A549细胞生长情况，结果表明，转染目的基因的实验组细胞较空病毒对照组以及空白对照组细胞增殖受到明显抑制。为明确AAV-Ang-2^shRNA^对A549细胞周期和凋亡的影响，我们采用流式细胞仪技术检测细胞周期和凋亡的变化。结果显示，实验组细胞较空病毒对照组以及空白对照组细胞出现G期阻滞和凋亡峰。上述结果表明，AAV-Ang-2^shRNA^有效抑制了肺腺癌A549细胞的体外增殖并诱导其调亡。

在体内实验中，AAV-Ang-2^shRNA^组裸鼠肿瘤细胞生长明显减慢，提示抑制Ang-2的表达可降低血管内皮细胞增殖、分化，使肿瘤生长速度减缓，但不能阻止肿瘤的发生。为进一步证实AAV-Ang-2^shRNA^在体内的作用，我们检测了RNA干扰后肿瘤组织中Ang-2及VEGF的表达情况。结果提示Ang-2靶向RNA干扰后肿瘤组织内Ang-2表达量明显降低，但对VEGF的表达没有明显的影响。说明实验组肿瘤生长减慢可能主要与Ang-2的抑制有关，而并不是由于VEGF的表达改变而引起。MVD是利用针对血管内皮细胞抗原的免疫组化技术对肿瘤微血管进行定量，可直接量化肿瘤的血管生成程度，较客观地反映肿瘤微血管形成的强度与肿瘤的侵袭性。本研究中，AAV-Ang-2^shRNA^组肿瘤MVD明显减小，说明该组肿瘤血管生成数目明显减少，不能满足新生肿瘤生长和侵袭的需要。这也可能是该组肿瘤生长缓慢的原因之一。肿瘤的发生不仅与细胞的异常增殖和分化有关，也与细胞凋亡的异常有关。本研究结果显示AAV-Ang-2^shRNA^组肿瘤组织内发现大量凋亡细胞，一方面可能是肿瘤新生血管减少引起的局部缺血、缺氧，肿瘤细胞增殖受到抑制、细胞凋亡增加所致，另一方面可能启动了肿瘤凋亡基因，其确切机制还有待进一步研究。细胞增殖核抗原PCNA是反映细胞增殖活性的常用指标，本研究结果提示，AAV-Ang-2^shRNA^组PCNA标记指数明显低于对照组，说明RNA干扰Ang-2对体内肿瘤的增殖能力有抑制作用。

本研究表明AAV-Ang-2^shRNA^可有效下调人肺腺癌A549细胞中Ang-2基因和蛋白表达，从而抑制细胞增殖。同时通过荷瘤裸鼠在体实验进一步证明Ang-2沉默可抑制A549细胞的生长、增殖，促进其凋亡，从而延缓肿瘤生长速度。Ang-2在肿瘤的血管生成和肿瘤进展中具有复杂功能。目前已经认识到，Ang-2通过Tie-2依赖的传导通路及整合素介导的信号传导，刺激肿瘤的血管生成、侵袭和转移。但是肿瘤组织中新生血管的形成是多因子协同的结果，Ang-2在肿瘤调控网络中的具体作用机制等问题都有待进一步研究。
